# Activación muscular del vasto lateral y del medial durante saltos con una sola pierna en los planos frontal y sagital en mujeres deportistas

**DOI:** 10.7705/biomedica.4938

**Published:** 2020-03-30

**Authors:** Andrés Felipe Villaquirán, Diana María Rivera, Enmanuel Fernando Portilla, Sandra Jimena Jácome

**Affiliations:** 1 Grupo de Movimiento Corporal Humano y Calidad de Vida, Universidad del Cauca, Popayán, Colombia Universidad del Cauca Grupo de Movimiento Corporal Humano y Calidad de Vida Universidad del Cauca Popayán Colombia; 2 Grupo de Salud y Motricidad Humana, Universidad del Cauca, Popayán, Colombia Universidad del Cauca Grupo de Salud y Motricidad Humana Universidad del Cauca Popayán Colombia

**Keywords:** electromiografía, deportes, mujeres, rodilla, músculos, ligamento cruzado anterior, potencia, prevención primaria, Electromyography, sports, women, knee, muscles, anterior cruciate ligament, potency, primary prevention

## Abstract

**Introducción.:**

Las diferencias en la activación muscular de las porciones lateral y medial del cuádriceps durante la ejecución del salto, pueden convertirse en un factor de riesgo neuromuscular al aumentar el valgo dinámico de rodilla y, en consecuencia, el riesgo de lesión del ligamento cruzado anterior de la rodilla.

**Objetivo.:**

Determinar la diferencia en la activación de los vastos lateral y medial del cuádriceps mediante electromiografía de superficie durante el salto con una sola pierna en los planos sagital y frontal en mujeres deportistas.

**Materiales y métodos.:**

Se hizo un estudio cuantitativo de corte transversal con la participación de 64 mujeres deportistas a quienes se les tomaron las medidas antropométricas. Se hicieron pruebas de salto vertical y lateral con una sola pierna evaluados mediante la activación electromiográfica de los vastos medial y lateral, y la valoración de la flexibilidad de la banda iliotibial.

**Resultados.:**

Se encontró una relación estadísticamente significativa (p≤0,05) entre el índice de masa corporal, el porcentaje de grasa y la potencia en los saltos verticales con una sola pierna. Se encontró, asimismo, significación estadística (p≤0,05) por una mayor activación del vasto lateral en el salto vertical con la pierna derecha y en el salto lateral con las dos piernas.

**Conclusión.:**

Las deportistas presentaron diferencias en la activación de los cuádriceps, siendo mayor la activación del vasto lateral en la mayoría de los saltos con una sola pierna tanto en el plano sagital como en el frontal, lo cual puede contribuir a un aumento del riesgo de lesión de rodilla en la práctica deportiva.

## Abstract (en)

**Introduction:**

Differences in the lateral and medial portions from quadriceps muscular activation during the execution of the jump can become a neuromuscular risk factor raising knee dynamic valgus and increasing the risk for anterior cruciate ligament injury.

**Objective:**

To determine the difference in the activation of the lateral and medial vastus using surface electromyography during single-leg jumps in the sagittal and frontal plane in female athletes.

**Materials and methods:**

This was a quantitative, cross-sectional study. A total of 64 female athletes participated. We carried out anthropometric measurements, vertical and lateral single-leg jump tests with the evaluation of vastus medialis and lateral electromyographic activation during the tests and iliotibial band flexibility assessment.

**Results:**

There was a statistically significant relationship (p≤0.05) between the body mass index, fat percentage, and vertical single-leg jump power. We also found statistical significance (p≤0.05), with greater activation of the lateral vastus in the right lower limb vertical jump and in the lateral jump in both lower limbs.

**Conclusion:**

The athletes presented differences in quadriceps activation, showing higher lateral vastus activation in most of the single-leg jumps, both in the sagittal and frontal plane. This may increase the risk for suffering knee injuries during sports practice.

Las lesiones de las extremidades inferiores son muy comunes en deportes de alto rendimiento ^1^ y, entre estas, se destacan las del ligamento cruzado anterior de la rodilla, las cuales varían según el sexo de los deportistas, el tipo de deporte, la frecuencia de competición y el nivel de los deportistas ^2^. Dichas lesiones son más frecuentes entre las atletas ^3^, con una tasa aproximada de lesión por exposición de 1,6 veces ^2^, siendo las principales aquellas sin contacto (60 %) ^4^. Además, el riesgo de lesión aumenta 1,8 veces cuando las deportistas no realizan ningún tipo de entrenamiento neuromuscular preventivo ^5^.

Este tipo de lesión se presenta con mayor frecuencia en deportes que involucran saltos, aceleraciones y desaceleraciones, y cambios de dirección ^6^^,^^7^. Se han establecido varios factores de riesgo intrínsecos y extrínsecos, entre ellos, el índice de masa corporal, los déficits biomecánicos y neuromusculares, la fatiga, la condición climática, el calzado deportivo, el nivel de competición, la superficie de juego y las condiciones climáticas ^7^.

Entre los factores de riesgo intrínsecos, se encuentran variantes anatómicas como un surco intercondilar estrecho, un ángulo p aumentado y una mayor rotación tibial lateral ^8^. Por otra parte, los factores de riesgo neuromusculares y biomecánicos, como la presencia de una mayor activación del cuádriceps que de la musculatura isquiosural, el déficit en la estabilidad muscular del tronco y de la cadera, un aumento del valgo de rodilla, la alteración de la estabilidad postural y los desequilibrios en la activación de los músculos mediales y laterales del cuádriceps y de los isquiosurales, entrañan un factor preponderante de riesgo de lesiones en el ligamento cruzado anterior y en las extremidades inferiores ^9^.

Para la planificación y elaboración de planes de prevención de lesiones deportivas, van Machelen propone en su modelo determinar los mecanismos de la lesión y su etiología para tomar mejores decisiones ^10^. Entre las pruebas propuestas para determinar los factores de riesgo de lesiones en las extremidades inferiores, está el salto vertical con una sola pierna ^1^, el cual es muy común y se repite continuamente en los entrenamientos y las competencias en diferentes deportes. Este salto es una actividad compleja, en la cual la fuerza de reacción del suelo es mayor, el ángulo de flexión máxima de la rodilla es menor, hay un mayor valgo de la rodilla en el contacto inicial y una menor velocidad angular, en comparación con el salto con dos pies ^11^.

Por otra parte, cabe destacar que los diferentes movimientos deportivos no se realizan en un solo plano y que la práctica del deporte implica una combinación de fuerzas verticales y horizontales muy importante para predecir el rendimiento y evaluar las asimetrías ^12^. Asimismo, la ejecución de un salto con cambio de dirección después de la fase de aterrizaje, entraña una mayor dificultad de respuesta mecánica de la rodilla. Además, el salto lateral puede considerarse como una maniobra mucho más riesgosa debido a las variantes en la respuesta neuromuscular que trae consigo esta acción. Sin embargo, son pocos los estudios que incluyen la evaluación del salto lateral con una sola pierna, una actividad con un alto grado de complejidad biomecánica, por lo que es necesario valorarla y entrenar adecuadamente al deportista, ya que así podrá planear con anticipación los patrones de movimiento requeridos en el momento de la competencia, reduciendo así el riesgo de lesión ^13^.

En cuanto a la banda iliotibial, esta actúa como uno de los estabilizadores laterales de la rodilla y, en sinergia con el ligamento cruzado anterior, en el control de la traslación anterior de la tibia ^14^ y como ayuda en la trasmisión de las fuerzas de la fascia toracolumbar a la rodilla ^15^. En consecuencia, su acortamiento constituye un factor clave en las diferentes disfunciones de las extremidades inferiores. Por ejemplo, la mala alineación de la rótula causa un aumento de su deslizamiento lateral sobre el fémur, lo que, a su vez, genera un retraso en la activación de los músculos de la cadera y de la rodilla, especialmente en movimientos anteroposteriores, aumentando así la probabilidad de padecer el síndrome de dolor patelofemoral ^16^.

La electromiografía de superficie utilizada en este estudio permite evaluar la función neuromuscular en acciones dinámicas y facilita la valoración de la activación y los desequilibrios que pueda presentar la musculatura en un determinado gesto deportivo ^17^. En ese sentido, es una herramienta importante para determinar las alteraciones en el patrón de activación entre el vasto lateral y el medial del cuádriceps ^18^, situación señalada como un factor de riesgo neuromuscular modificable para la lesión del ligamento cruzado anterior ^7^^,^^9^.

En este contexto, y dada la importancia del comportamiento de la activación muscular del cuádriceps como factor de riesgo neuromuscular en este tipo de lesión, y los pocos estudios de medición electromiográfica durante el salto con una sola pierna realizados en la región, en este se propuso determinar la diferencia en la activación del vasto lateral y del medial del cuádriceps durante el salto en el plano sagital y frontal en mujeres deportistas mediante electromiografía de superficie. Además, el interés del equipo investigador se centró en determinar las posibles relaciones entre las características deportivas y antropométricas, y la potencia del salto en este grupo poblacional.

## Materiales y métodos

### Diseño del estudio y muestra

Se llevó a cabo un estudio cuantitativo de corte transversal y diseño correlacional durante el 2018. La muestra se seleccionó por conveniencia e incluyó a 64 mujeres deportistas en Popayán, que habían participado activamente, por lo menos, durante el año anterior en competencias nacionales e internacionales en su respectivo deporte (taekwondo, fútbol, fútbol sala, yudo, halterofilia, patinaje, voleibol).

Cabe aclarar que, a pesar de la diferencia en la mecánica del rendimiento muscular propia de cada deporte, toda preparación deportiva incluye el salto como una tarea motriz básica e importante para el desarrollo de la potencia muscular.

Según el número de participantes, la forma de juego y los elementos comunes utilizados para la competencia, estas disciplinas se clasifican como deportes de tiempo y marca, deportes de pelota y raquetas, y deportes de combate.

Como criterios de inclusión, se tuvieron en cuenta los siguientes aspectos: ser mayor de 16 años, hacer parte de un seleccionado deportivo con participación nacional en el deporte y categoría correspondientes, y aceptar la participación voluntaria en el estudio. Se excluyeron aquellas deportistas que no habían tenido competencia a nivel nacional durante el último año, aquellas que presentaban una lesión que les impedía realizar las pruebas, así como las que estaban en proceso de rehabilitación deportiva o quienes reportaron haber sido dadas de alta de la actividad competitiva tres meses antes de la evaluación.

### Procedimiento

Después de explicar los objetivos del estudio, el procedimiento de la evaluación, así como los beneficios y las posibles complicaciones durante las pruebas, los derechos de las participantes y los demás aspectos éticos del estudio, se les solicitó la firma del consentimiento informado.

El proceso de valoración se inició con una encuesta para tomar los datos sociodemográficos (edad, sexo) y deportivos (años de práctica, experiencia, horas diarias de práctica, frecuencia semanal de práctica, lesiones previas, dominancia), la cual había sido ajustada previamente mediante una prueba piloto. La dominancia se clasificó según el índice de asimetría midiendo el rendimiento de las extremidades inferiores en los saltos con una sola pierna y tomando como referencia una diferencia entre los miembros inferiores de 10 a 15 % ^19^.

A continuación, se hizo la valoración antropométrica (índice de masa corporal, sumatoria de pliegues y porcentaje de grasa), tomando como referencia las recomendaciones de la *International Society for the Advancement of Kinanthropometry,* ISAK ^20^. Para la clasificación del índice de masa corporal (IMC), se usaron los parámetros establecidos por la Organización Mundial de la Salud (OMS) ^21^. Para complementar la evaluación, dado que el IMC no es una medida que permita determinar la distribución de la masa corporal, se calculó el contenido de grasa mediante la sumatoria de pliegues ^22^, el cual es un índice de adiposidad individual y un indicador del aumento de la masa grasa. El porcentaje de grasa se determinó, además, mediante la fórmula propuesta por Yuhasz ^23^.

Se hizo un calentamiento estandarizado con todas las atletas sobre un tapiz rodante a una velocidad de 8 km/hora durante 8 minutos. A continuación, se colocaron los electrodos de superficie adhesivos de botón a una distancia de 1 cm entre electrodos en el vasto medial y en el lateral de los cuádriceps, tomando como referencia el protocolo del proyecto *Surface ElectroMyoGraphy for the Non-Invasive Assessment of Muscles* (SENIAM) ^24^. El electrodo de referencia se ubicó en la apófisis estiloides cubital de la muñeca derecha. Se obtuvo la contracción voluntaria máxima para la normalización de los trazados pidiendo a las deportistas tres contracciones isométricas máximas de 6 segundos de duración.

En la fase de registro, después de recibir las instrucciones, las deportistas realizaron los saltos verticales y laterales correspondientes a cada prueba. Los registros electromigráficos se hicieron con un electromiógrafo inalámbrico de superficie MYON 320 (Biomec), con una velocidad de muestreo de 4.000 Hz por canal, frecuencia de transmisión de 2,4 GHz y una latencia constante de 16 m.

Para procesar la señal, se eliminaron los artefactos y cualquier tipo de contaminación del registro con los filtros pasa bajo y pasa alto y el eliminador de banda. Se rectificó la señal y, por último, se aplicó el algoritmo de suavizado de la raíz de la media cuadrática *(root mean square,* RMS), el cual rectifica la señal y aplica un suavizado que permite estudiar el parámetro de amplitud como indicador de la actividad muscular, con el objetivo de obtener una imagen más próxima a la activación muscular y más fácil de observar. Los valores se normalizaron con respecto a la contracción voluntaria máxima.

Posteriormente, se hicieron las pruebas de salto vertical y lateral con una sola pierna sobre una plataforma de contacto Axon Jump, modelo S, y el programa Axon Jump, versión 4.2. En cuanto al salto vertical con una sola pierna, las deportistas se apoyaban en una sola extremidad sobre la plataforma con las manos en la cadera para después flexionar la cadera y la rodilla aproximadamente a 90 grados y hacer un salto vertical tan alto como les fuera posible ^25^. Para el salto lateral, las atletas se paraban apoyadas en una sola pierna sobre la plataforma de contacto con las manos en la cadera y flexionaban la cadera y la rodilla a 90 grados para hacer un salto lateral tan alto y lejos como les fuera posible ^26^.

Por último, para la evaluación de la flexibilidad de la banda iliotibial, se usó la maniobra de Ober ^27^ con la participante en decúbito lateral y el evaluador ubicado detrás de la persona; este fija con una mano la cadera correspondiente y, con la otra, la flexiona y la abduce dejando caer lentamente la extremidad hasta el tope permitido por la participante. Sobre la pierna evaluada se colocó un goniómetro electrónico operado con el programa Unicore de Mobee Med™ para cuantificar la posición final. Cuando la deportista no alcanzaba los cero grados (0^0^) sobre la horizontal, se consideraba como positivo el resultado de la maniobra ^28^.

### Aspectos éticos y legales

La presente investigación se llevó a cabo bajo los parámetros y recomendaciones establecidas para la investigación en humanos en la Declaración de Helsinki ^29^ y la Resolución 8430 del Ministerio de Salud de Colombia ^30^, según la cual este estudio se cataloga como de riesgo mínimo. Se contó con la aprobación del Comité de Ética y la inscripción del proyecto en el Sistema de Investigaciones de la Universidad del Cauca (código 4925).

### Análisis estadístico

En el análisis descriptivo, se emplearon distribuciones de frecuencia y, para las variables numéricas, porcentajes y medidas de tendencia central y dispersión. El análisis inferencial se hizo mediante las pruebas paramétricas Anova y t de Student después de verificar los requisitos de distribución de normalidad (prueba de Shapiro-Wilk con significación mayor de 0,05) y la homogeneidad de varianzas (prueba de Levene con significación mayor de 0,05); también, se hizo la prueba no paramétrica U de Mann-Whitney para las variables cualitativas y aquellas cuantitativas que no cumplieron con los requisitos. Asimismo, se utilizaron correlaciones bivariadas entre las variables cuantitativas ordinales. En todas las pruebas de dos colas, el nivel de significación se estableció como p menor de 0,005.

## Resultados

La edad promedio de las participantes fue de 20,73 ± 4,73 años, con una media de entrenamientos semanales de 4,2 ± 1,39 días, un promedio de 2,37 ± 0,89 horas diarias de práctica y una experiencia deportiva en años de 6,78 ± 4,7 según lo refirieron ellas mismas. En cuanto a las variables antropométricas, su IMC era de 22,25 ± 2,0 kg/m^2^, el porcentaje de grasa de 18,32 ± 3,72, y solo el 7,9 % tenía sobrepeso. El 88,9 % de las participantes tenía dominancia derecha, y el 21 % reportó haber sufrido alguna lesión de rodilla durante su carrera deportiva.

En cuanto a los saltos, la altura promedio con la pierna derecha en el salto vertical fue de 12,35 ± 3,99 cm, en tanto que con la izquierda fue de 12,27 ± 3,62 cm. En el salto lateral, el promedio en la altura fue de 10,95 ± 3,70 cm con la pierna derecha y de 10,35 ± 3,20 cm con la izquierda.

**Figura 1:**
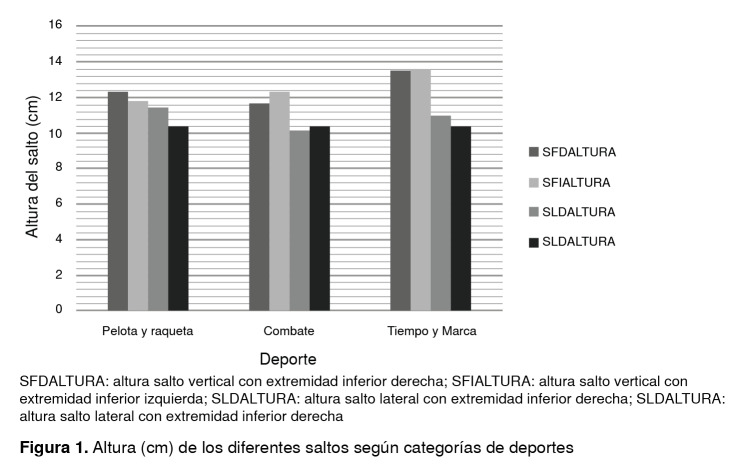
Altura (cm) de los diferentes saltos según categorías de deportes

SFDALTURA: altura salto vertical con extremidad inferior derecha; SFIALTURA: altura salto vertical con extremidad inferior izquierda; SLDALTURA: altura salto lateral con extremidad inferior derecha; SLDALTURA: altura salto lateral con extremidad inferior derecha

Además, quienes practicaban deportes de tiempo y marca presentaron mayores promedios de altura en el salto vertical, en tanto que aquellas dedicadas a deportes de pelota y raqueta presentaron medias superiores en el salto lateral; sin embargo, estas diferencias no fueron estadísticamente significativas (p>0,005) (figura 1).

Con respecto a la altura de los saltos, las características antropométricas y los años de práctica, se encontró una significación estadística entre el salto lateral con la pierna derecha y los años de práctica, con una correlación positiva débil, es decir que, a mayor experiencia deportiva, mayor fue la altura del salto. En los saltos verticales con una sola pierna y el salto lateral con la pierna izquierda, hubo relación significativa entre el porcentaje de grasa y el IMC, aunque con correlaciones débiles, lo cual sugiere que, a mayor grasa o IMC, menor fue la altura del salto (cuadro 1).

**Cuadro 1:** Correlación entre la altura de los saltos y las características antropométricas y años de práctica de las participantes

Media	Media/desviación estándar	Características deportivas	Significación	Correlación
	(cm)	y antropométricas		
Salto lateral derecho	10,95 ± 3,72	Años de práctica	0,020	0,2
		Porcentaje de grasa	0,021	-0,3
Salto vertical derecho	12,35 ± 3,99	IMC	0,001	-0,3
		Porcentaje de grasa	0,000	-0,3
Salto vertical izquierdo	12,27 ± 3,62	IMC	0,008	-0,2
		Porcentaje de grasa	0,010	-0,3
Salto lateral izquierdo	10,35 ± 3,20	Porcentaje de grasa	0,039	-0,2
				

IMC: índice de masa corporal

**Figura 2:**
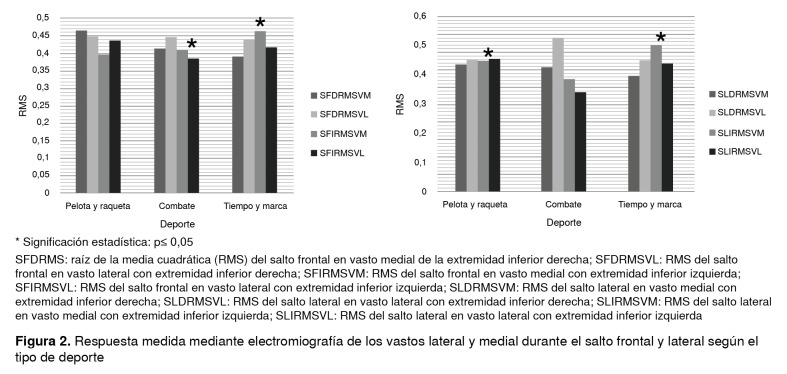
Respuesta medida mediante electromiografía de los vastos lateral y medial durante el salto frontal y lateral según el tipo de deporte

**Figura 3:**
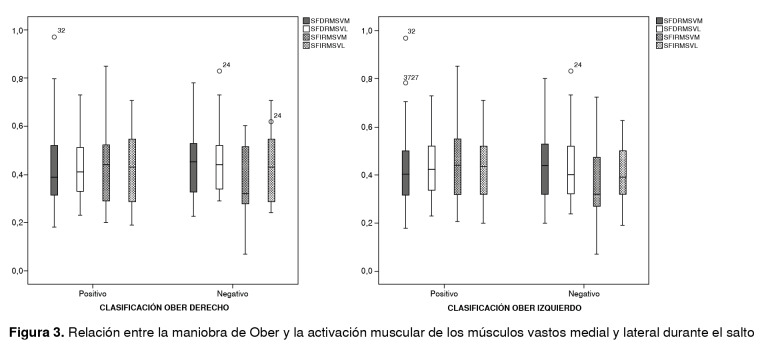
Relación entre la maniobra de Ober y la activación muscular de los músculos vastos medial y lateral durante el salto

En cuanto a la activación muscular durante los saltos, se encontró una diferencia estadísticamente significativa al registrarse una mayor activación del vasto lateral en el salto vertical con la pierna derecha y en el lateral con las dos piernas (cuadro 2). Por otra parte, se encontraron diferencias estadísticas en la activación muscular en el salto vertical entre los deportes de combate y los de tiempo y marca, y entre las participantes dedicadas a los deportes de pelota y raqueta y a los de tiempo y marca en el salto lateral (figura 2). Además, no se encontraron diferencias estadísticamente significativas en la activación del vasto lateral entre las deportistas con registros normales y aquellas con disminución del arco de movimiento en la maniobra de Ober (figura 3).

**Cuadro 2:** Respuesta medida mediante electromiografía del vasto medial y el lateral de las extremidades derecha e izquierda durante los saltos frontal y lateral de las deportistas

	Media	Media/desviación	Significación
		estándar (µV)	
Salto frontal	Extremidad inferior derecha		
	Vasto medial	0,433 ± 0,160	0,003
	Vasto lateral	0,445 ± 0,143	
	Extremidad inferior izquierda		
	Vasto medial	0,413 ± 0,162	0,197
	Vasto lateral	0,417 ± 0,132	
			
Salto lateral	Extremidad inferior derecha		
	Vasto medial	0,423 ± 0,143	0,014
	Vasto lateral	0,469 ± 0,214	
	Extremidad inferior izquierda		
	Vasto medial	0,442 ± 0,164	0,072
	Vasto lateral	0,420 ± 0,140	
			

* Significación estadística: p≤ 0,05 SFDRMS: raíz de la media cuadrática (RMS) del salto frontal en vasto medial de la extremidad inferior derecha; SFDRMSVL: RMS del salto frontal en vasto lateral con extremidad inferior derecha; SFIRMSVM: RMS del salto frontal en vasto medial con extremidad inferior izquierda; SFIRMSVL: RMS del salto frontal en vasto lateral con extremidad inferior izquierda; SLDRMSVM: RMS del salto lateral en vasto medial con extremidad inferior derecha; SLDRMSVL: RMS del salto lateral en vasto lateral con extremidad inferior derecha; SLIRMSVM: RMS del salto lateral en vasto medial con extremidad inferior izquierda; SLIRMSVL: RMS del salto lateral en vasto lateral con extremidad inferior izquierda

## Discusión

El control en la activación muscular en los movimientos de los deportistas puede reducir el riesgo de lesiones. La estabilización dinámica de la articulación de la rodilla con un adecuado control neuromuscular mediante acciones musculares coordinadas y una adecuada coactivación durante los saltos, cortes y giros, puede proteger la articulación ^31^. En las mujeres, se presentan más factores de riesgo con un incremento en la aducción de cadera ^32^ y la rotación interna de fémur y una reducción de la flexión de rodilla ^33^ y del valgo dinámico ^34^ durante las maniobras combinadas en diferentes planos y los procesos de desaceleración ^35^. Además, se han relacionado otros factores con la potencia del salto, tales como la composición corporal y la flexibilidad de la banda iliotibial.

En cuanto a la activación muscular del vasto medial y el lateral, en este estudio se encontraron diferencias estadísticamente significativas entre los dos vientres musculares, siendo el vasto lateral el que mayor activación presentó en el plano sagital durante el salto con la pierna derecha y en los saltos frontales con las dos extremidades. En este sentido, Palmieri-Smith, *et al.,* encontraron una relación entre la presencia del valgo de rodilla y una mayor activación del vasto lateral ^36^. Por su parte, en su evaluación de la activación de la musculatura del cuádriceps durante una posición que simula un riesgo alto de lesión del ligamento cruzado anterior, Myer, *et al.,* reportaron una disminución de la activación del vasto medial en mujeres, lo que genera un incremento de la carga y la fuerza de cizallamiento anterior sobre dicho ligamento ^37^. Malfait, *et al.,* registraron una mayor activación del vasto lateral y porción lateral de la musculatura isquiosural en fase de máxima carga en el aterrizaje del salto vertical, lo que generó una mayor extensión de la cadera y de la flexión externa de rodilla con momentos de abducción durante el aterrizaje ^38^. Asimismo, Vanmeerhaeghe, *et al.,* encontraron que los desequilibrios en la activación de los músculos mediales y laterales de los cuádriceps constituyen factores neuromusculares de riesgo de lesión de la extremidad inferior ^9^.

Si bien este patrón puede aumentar el valgo dinámico de rodilla, no es el único factor modificable para la lesión sin contacto del ligamento cruzado anterior. Según el consenso para la prevención de las lesiones de rodilla realizado en el 2017 por la *German Knee Society* (GKS), un mal control de la cadera, la debilidad de los flexores de rodilla y de los abductores de cadera, el déficit propioceptivo, un estado físico deficiente, y un control insuficiente del tronco y la cadera, también están asociados con un mayor riesgo de lesión ^39^ y deben tenerse en cuenta para complementar futuros estudios en mujeres deportistas, pues su control sería de gran utilidad para adoptar mejores decisiones en los programas de entrenamiento neuromuscular, dado su comprobado papel en la disminución del riesgo de lesión del ligamento cruzado anterior ^40^.

Por otra parte, muchas de las maniobras deportivas requieren una combinación de planos y fuerzas horizontales, verticales y laterales, sobre todo en los deportes de velocidad y agilidad, por lo cual en este estudio se evaluó mediante electromiografía la activación durante los saltos laterales, los cuales pueden predecir de mejor forma el rendimiento en los deportes con cambios de dirección y movimientos que influyen claramente en el plano frontal ^13^. En este estudio se encontró un desequilibrio entre los vastos en los saltos laterales, en tanto que, en el de Jenkins, *et al.,* se registraron respuestas en valgo y en varo en la angulación y la velocidad de desplazamiento de la rodilla en el plano frontal durante el salto en las mujeres ^41^. Además, se ha encontrado que el comportamiento de la rodilla en el plano frontal depende de los abductores, los rotadores externos, los extensores de cadera y los músculos centrales (abdomen, espalda, pelvis), componentes que pueden generar el desplazamiento de la rótula o la rotación medial femoral, o aumentar el ángulo Q ^42^.

Con relación a la composición corporal (IMC y grasa corporal) y la potencia del salto, en el presente estudio se encontró una disminución de la altura del salto a medida que el IMC y el porcentaje de grasa aumentaban en las deportistas evaluadas. En este sentido, en su estudio con mujeres jugadoras de voleibol, Nokolaidis encontró una relación inversa entre el IMC y el porcentaje de grasa y la potencia del salto, así como con otras variables de la condición física de las atletas ^43^. En un estudio realizado en jugadoras de waterpolo, se demostró que los movimientos de potencia y velocidad dentro del agua los realizan con mayor eficiencia las jugadoras con valores normales de IMC y grasa corporal ^44^. Sin embargo, González-Rave, *et al.,* encontraron que no había una relación directa entre los cambios de la composición corporal y la ganancia de fuerza y potencia durante un programa de entrenamiento de 24 semanas para jugadoras profesionales de voleibol ^45^, resultados que difieren de lo encontrado en el presente estudio. El estudio de estas variables permitirá, sin embargo, que los entrenadores y profesionales de la salud ajusten los programas de entrenamiento para el desarrollo de la mejor forma deportiva y la prevención de lesiones.

En cuanto a la evaluación de la flexibilidad de la banda iliotibial con la maniobra de Ober, no se encontraron diferencias estadísticamente significativas entre las deportistas que presentaron una disminución del arco de movimiento y la activación del vasto lateral durante las pruebas, aunque se ha reportado un retraso en la latencia del vasto lateral y del medial en acciones con alteraciones repentinas en el equilibrio, situación que podría provocar un déficit en el control neuromuscular de la cadera y la rodilla, y aumentar el riesgo de lesiones ^16^.

En conclusión, se encontró un desequilibrio en la activación muscular de los cuádriceps durante los saltos verticales y laterales realizados por las participantes en el estudio, pero no se encontró relación entre la activación del vasto lateral y la disminución de la flexibilidad de la banda iliotibial. Además, en el estudio se encontró que el incremento del IMC y del porcentaje de grasa puede afectar negativamente la potencia del salto. Estos resultados permiten a los entrenadores y al personal de salud tomar decisiones más adecuadas en la planeación y ejecución de programas de prevención de lesiones de rodilla en mujeres deportistas.

Por último, el estudio tuvo sus limitaciones, ya que solo se evaluó mediante electromiografía el comportamiento del músculo del cuádriceps durante los movimientos. Por esta razón, se sugiere incluir en próximos estudios la coactivación del cuádriceps y los isquiosurales, así como un análisis biomecánico en 2D o 3D durante los saltos con una sola pierna para complementar estos resultados. Asimismo, se sugiere hacer nuevos estudios que complementen la evaluación del salto con otras tareas específicas de cada deporte para evaluar el comportamiento muscular durante acciones que requieren de la potencia de las extremidades inferiores para, así, establecer las diferencias de cada disciplina deportiva. De todas maneras, el estudio puede ser de gran utilidad en la evaluación de los factores de riesgo neuromuscular que afectan el rendimiento deportivo y alteran la salud de las atletas.
